# What we know about the trends, prospects, and challenges of human resource outsourcing: A systematic literature review

**DOI:** 10.1016/j.heliyon.2023.e19018

**Published:** 2023-08-07

**Authors:** Md. Nazmus Sakib, Fariya Tabassum, Dr. Md. Mesbah Uddin

**Affiliations:** a Lecturer, Department of Management, University of Dhaka, Dhaka, 1000, Bangladesh; b Lecturer, Human Resource Management Discipline, Khulna University, Khulna, 9208, Bangladesh; c Associate professor, Department of Management, University of Dhaka, Dhaka, 1000, Bangladesh

**Keywords:** Human resource outsourcing, HRO, HR outsourcing, Systematic literature review, Post Covid HRO

## Abstract

This study aims to comprehensively review the literature on human resource outsourcing (HRO) published from 2001 to 2021. The study begins with metadata analysis on 69 papers and presents insights into 32 papers on HRO identified from the Scopus and ISI Web of Science databases. The literature is classified based on content analysis, which comprises conceptual understanding, drivers and barriers, functions outsourced, and firm performance. The study reveals that cost advantage, organisational learning, and the opportunity to concentrate on core business functions motivate the organisation to practice HRO. However, the lack of psychological contact among current employees, the risk of opportunism in the freelancing organisation, lack of management legislation, and prior experience are the common barriers to HRO adoption. Despite thesedrawbacks and barriers, recruitment, payroll processing, and technology-centric human resource (HR) activities are standard HR functions outsourced by organisations. The contributions of this study are to offer an integrated and conclusive definition of HRO and provide a simple, easy-to-understand, yet comprehensive framework for understanding HRO practices in any organisation. Researchers and academicians can utilize this paper to explore future research directions while gaining a thorough understanding of the HRO concept.

## 1 Introduction

As globalization continues to reshape the business landscape, the use of HRO services is gaining significant popularity among businesses. This trend is driven by several advantages, including cost savings, the ability to meet flexible employment requirements, risk reduction, and access to top-notch talent pools. By leveraging HRO services, businesses can optimize their operations while tapping into high-quality labor resources [[1], [2], [3]]. Similarly, the concept has become a buzzword in the discourse of business policymakers, researchers, and industrialists in recent years [1,3]. Although HRO practices are increasing, organisations are not able to use it to the same extent; hence the strategy and structures matter [4]. However, there is a growing debate among policymakers about whether HRO is an opportunity or risk for an organisation [1]. Earlier research argues both positive and negative aspects of HRO. A great deal of previous studies argues that in the age of globalization, firms in developing countries can be immensely benefitted from outsourcing HR functions from the viewpoint of their strategic aspects. Previous studies claim that the strategic decision to outsource HR functions aims to minimize costs in situations where in-house expertise is lacking, leading to the outsourcing of both core and non-core HR functions, particularly beneficial in uncertain demand scenarios [[5], [6], [7], [8], [9]]. A study by Coggburn [6] also argues similarly in favor of cost efficiency that when the external vendor provides the same service at a lower cost than the in-house service maker, it is called cost-effectiveness. On the contrary, earlier studies have identified opposing arguments against organisations’ decisions to outsource HR, highlighting potential drawbacks and limitations [1]. The authors argue that HRO leads to a reduction of employees and impels to recruit seasonal employees. Therefore, there would be a gap in organisational and interpersonal relationships, encouraging a lack of psychological contact and destroying pre-existing networks and organisational memory. In the long run, the client company becomes inefficient, loses its organisational values, fails to develop essential knowledge, and lacks unique competencies [1]. Despite having positive and negative arguments, there has been a modest growth rate rather than overwhelmingly fleet since the 1990s, and the practice of HRO is anticipated to have future upliftment.

However, the research on HRO has been increasing for the last couple of years. But as far as the knowledge of the authors, there are very few systematic literature reviews found on HRO. Although the number of review papers in this area is negligible, some authors look into critical factors on HRO [2,3]. A few papers focus on some selected issues of HRO [1]. Although a study by Yan [10] proposes a conceptual framework for examining the effectiveness of practicing HRO in ‘make or buy’ decisions, where metadata analysis is completely ignored. A study by Shen [11] highlights the significance of supporting the development of HRO practices by focusing on how to effectively implement HRM functions, the reasons for adopting this approach, and practical advice for managers in the field. Partially similar findings are found in the study by Cooke [12] which describes how outsourcing effectiveness is determined and affects business performance. Where the first paper emphasises the resource-based theory, while the second one acknowledges the significance of both resource-based theory and institutional theory in decision-making pertaining to the HRO concept. Furthermore, existing research also emphasises the strong connection between successful HR outsourcing and the quality of partnerships [13]. Considering only all the empirical studies of HRO, a study by Sim [14] employing a grounded coded technique, provides valuable insights into the field of HRO.

None of the review articles contributes to a metadata analysis that establishes the criteria for selecting articles, nor do they develop a theoretical framework for further research approaches. In contrast, most of the reviews contribute to the analysis of the evidence on HRO. In addition, earlier studies collect and analyse papers based on a subjective approach, potentially leading to bias. Therefore, there is a need to collect papers based on an objective approach. Hence, this paper has adopted an objective approach for data collection from the Scopus database, which objectively collects data based on search strings. While this searching method impartially limits keyword search to reliable sources, it does overlook less established sources [15]. Further, due to the increased use of and considerable debacles on HRO, there is a growing demand to review the concept thoroughly with its trends, challenges & limitations, along with the benefits that might be brought with the hand of HRO. Thus, the limited number of previous review papers justifies the need for the present study to encompass recent developments and provide key insights, which would contribute to the existing body of knowledge on HRO [16].

This study therefore addresses the following research questions: Who are the influential actors in HRO? What are the present trends in the HRO arena? What are the influential journals of HRO? What are the key contributing countries, institutions, affiliations, & subject areas of HRO? And what are the key themes that would help classify and arrange the existing literature on HRO & finally illustrate a simple framework to understand the concept of HRO? Hence, the main aim of this paper is to review the hitherto extant literature in the domain of HRO to acquire knowledge on the current trends and developments of HRO while identifying/discovering future research avenues.

The data for this paper has been collected from the Scopus and ISI Web of Science databases from 2001 to August 2021. Earlier studies have utilized these two databases as credible sources for conducting a systematic literature review paper [[17], [18], [19]]. For the systematic literature review, 69 articles from the Scopus and Web of Science databases were objectively analysed in this study. The metadata analysis depicts the descriptive statistics of the year of publications, influential authors, study countries, affiliations, funding sponsors, influential journals, and the study area of HRO. Therefore, the contributions of this paper are manifold: first, this study will offer an integrated and conclusive definition of HRO. Second, this study will present the key insights and trends of HRO, which are significantly different from earlier studies. Third, the paper will contribute a very simple, easy, understandable, but comprehensive framework of HRO. Finally, researchers and academicians would utilize this paper to explore future research directions while thoroughly understanding the HRO concept. The remainder of the paper begins by outlining the concept of HRO in section two. Section three presents the detailed methodology of this paper, while key insights, observations, concepts, and recommendations are presented in section four. Section five illustrates the insights into HRO through the lens of previous literature and section six discusses the implications of this study in academia and practices. Finally, section seven uncovers the conclusion and limitations of this study, along with future research avenues.

## 2 Human resource outsourcing

Academics and practitioners echo the same while considering the value of HRO practices to drag down the costs by adopting flexible employment demands, improving customer services, and maintaining global standardization [5,20,21]. Although it appeared as a concept more than two decades ago, scholarly contributions in this domain gained momentum in 2010, and since then, it has maintained an erratic growth trend. However, the idea maintained a steady growth till 2016.

Traditionally, outsourcing is considered a nonstrategic element where resources are left for significant strategic contribution [22]. In the past, outsourcing HR activities was based on a contractual agreement and trust between the vendor company and service receiver company, whereas today it is pursued as a means to gain a competitive advantage [13], with HRO influencing an organization's financial circumstances while also serving as a tool to drive innovation and enhance stakeholder performance [23]. Earlier scholars defined HRO that primarily focused on these key advantages. For instance, Butler [5] defines HRO and argues that HRO is what an organisation shares its human resource functions and transfers internal work responsibility to an outside supplier for getting HR related services. Whereas Zhao [24] takes a utilitarian perspective in defining HRO, emphasizing that it involves outsourcing HR centric tasks to external organisations. Despite undeniable interest from the stakeholder, research contributions in this area are still unfledged.The existing literature primarily focuses on examining the factors that influence the adoption of HRO. However, there is a lack of emphasis on clearly defining the term HRO from various perspectives. To address this gap, Appendix A summarises and presents all the potential definitions of HRO provided by researchers.

## 3 Method

This is a systematic literature review offering a deep dive into the domain of HRO following a rigorous scientific approach. The research methodology involves a systematic literature search and selection process of relevant academic articles, reports, and other publications related to HRO. The selected literature is then thoroughly analysed using qualitative techniques such as thematic and content analyses to identify common themes, patterns, and key insights related to the domain of HRO [25,26]. This study employs a meta-synthesis, which involves synthesizing the findings of multiple studies to generate overarching themes and theoretical frameworks. The rigorous scientific approach ensures that the review is comprehensive, objective, and grounded in the existing body of knowledge on HRO, providing valuable insights for practitioners, policymakers, and researchers in the field of HRO [27]. Therefore, reliable sources and systematic processes have been used to collect data for this study. According to the researchers, when conducting a systematic literature review, it is required to assess the different types of sources, for example, journal articles, books, and web-based resources [28,29]. However, an integrative literature review generates new ideas and directions for the specific field, laying a foundation for future research or theory [30]. Following the above conception, this study is engaged within four stages of screening data based on content analysis following the widely accepted process of the PRISMA model as suggested by Moher [31,32]. Initially, data has been collected from the Scopus database, considered a reliable data source [19,33,34].

### 3.1 Identification of the data

The Scopus integrated database served as the foundation for collecting data in this study, encompassing all key publishers, including Taylor and Francis, Emerald, Springer, Nature, Wiley, and Elsevier. The academician's contributions to HRO and related fields began in 2001. Consequently, a search was conducted to identify papers covering the period from 2001 to August 2021. The search string included terms such as “Human Resource Outsourcing,” “HR outsourcing,” “Personnel Management Outsourcing,” “Talent Management Outsourcing,” and “Human Capital Management Outsourcing,” limited to the article title, keywords, and abstract. Initially, a total of 135 results was shown, as displayed in Table 1.

**Table 1:** Appearance of papers by the initial search result.

Search string	Results (Number of articles)	Limit to
“Human Resource Outsourcing”	52	Article title, keywords, and abstract
“HR Outsourcing”	24	Article title, keywords, and abstract
“Personnel Management Outsourcing”	33	Article title, keywords, and abstract
“Talent Management Outsourcing”	12	Article title, keywords, and abstract
“Human Capital Management Outsourcing”	14	Article title, keywords, and abstract

### 3.2 Screening of initial data

During the initial data identification stage, a total of 135 items comprising articles, book chapters, conference papers, and reviews were selected. The selection process followed the recommendations outlined in previous literature [17,18]. Subsequently, non-article items were excluded, narrowing down the selection. After removing 66 duplicated papers, a final set of 69 papers remained for metadata analysis.

### 3.3 Eligibility determination

Following the suggestions of previous literature by Islam and Tseng [17,18], this study objectively determined 32 eligible papers by performing a keyword search in the ISI Web of Science database. The search was restricted to the titles of the papers using the keywords “human resource outsourcing,” “personnel outsourcing,” and “HR outsourcing.” The study incorporated papers published between 2001 and August 2021. Papers appearing in both the ISI Web of Science and Scopus databases were selected as eligible for the next step. It is worth noting that most papers in the ISI Web of Science database are also included in the Scopus database. In addition, the ISI Web of Science database was frequently used to gain insights from previous research [18,[35], [36], [37], [38]].

### 3.4 Data inclusion

**Fig. 1:**
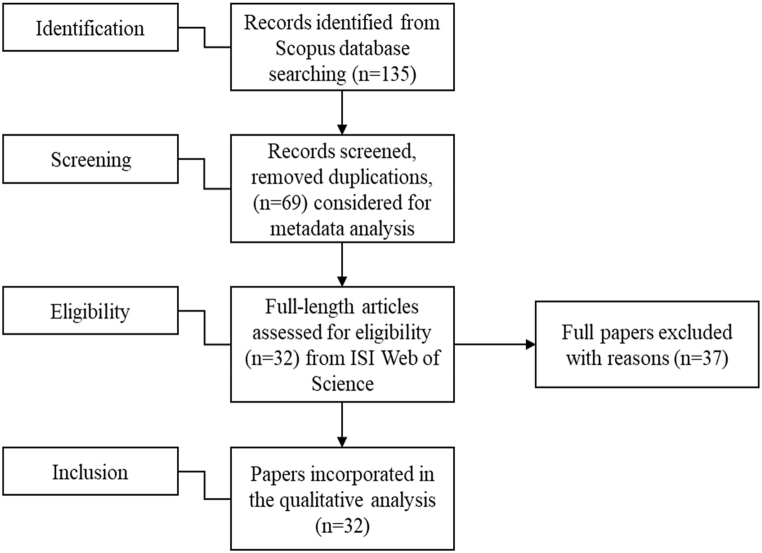
PRISMA method utilized in the study [31].

The paper includes 69 papers for metadata analysis from the Scopus database and 32 papers from the ISI Web of Science database to gain insights and guide future research directions (see Fig. 1). It is worth mentioning that these two databases are appropriate for generalizability due to their indexing of journals from other significant databases, such as Science Direct, Emerald, PLOS, Elsevier, Wiley, Springer, IGI Global, Inderscience, Taylor and Francis, among others [18].

Previous papers analysed the data and presented insights, being shortlisted by subjective judgment. This paper, however, selects 32 papers objectively from influential journals, following the four steps highlighted in Fig. 1. The study ensures that the data is sourced from an enriched and standard database while maintaining unbiasedness. The paper collects data exclusively from the ISI Web of Science database through a keyword search. In addition, it is worth noting that the papers found in ISI Web of Science may also be found in the Scopus database.

## 4 Analysis and observations

The metadata analysis and insights have been presented in the section below. Metadata analysis has been performed based on 69 papers, and the key insights have been derived from content analysis of 32 papers.

### 4.1 Metadata analysis

This section presents descriptive statistics that uncover the metadata analysis of 69 papers. The analysis includes the names of journals, years, authors, funding institutions, citations, countries, subject areas, and affiliated institutions for the 69 papers. There is a possibility of counting a single paper multiple times in the metadata analysis; for example, if a paper is counted based on its authors, it is also counted in terms of its year and area. In some instances, this study does not present the statistics in an exhaustive list but instead uses a reduced format to enhance readability.

#### 4.1.1 Publications by year

The exploration of HRO in academic papers began in early 2001, with two notable publications in this field. However, academic contributions pertaining to HRO were relatively scarce until the year 2010. In 2010, the maximum number of papers was published, totaling 16. Both in 2011 and 2015, seven papers were found, indicating significant progress. In 2014 and 2016, the number of articles stood at six for each year, respectively.

#### 4.1.2 Publication by journals

Although the literature on HRO is found in diversified journals, three papers each are sourced from both the Journal of Business Research and the International Journal of Human Resource Management. Additionally, six papers are obtained from Advanced Science Letters, Human Resource Management, and Human Resource Management International Journal, with two papers from each journal. The meta-analysis reveals that the top authors who have made contributions to HRO-related papers are ranked based on Scopus-indexed journals.

Abu Noor Hidayah emerges as the most influential author in this sector, having contributed to four papers. Furthermore, three authors, namely Bi, X.Q., Mohd Fitri Mansor, and Zhou, Q.X., have each contributed to three papers. Abdul-Halim Hasliza has contributed to two papers related to HRO. The remaining authors have each contributed to a single paper.

#### 4.1.3 Publications by country/territory

The practice of HRM in China has undergone significant changes due to heightened business competition, both domestically and internationally, and banal cultural values are contributing to the configuration of HR practice [24]. As a result, China dominates the production of papers related to HRO, accounting for 35% of the total. However, Malaysia and the United States both share the same percentage, contributing 16% of the total papers each. Similarly, Taiwan and the United Kingdom have each contributed 7% of the papers. Furthermore, two Asian countries (India and Hong Kong) and North America (Canada) have produced 12% of the total HRO-related papers, with each country contributing 4%. On the other hand, Australia has the lowest number of HRO related papers, while Vietnam is the second-to-last contributing country, producing 3% of the total.

It is evident that Asian countries are prolific in practicing HRO. This might be attributed to the cost-cutting tendency in Asian workplace, culture and technological advancements.

#### 4.1.4 Publication by document type

While it is true that HRO is not commonly discussed in academia, reflecting a limited number of book chapters and review papers,a large portion of 58% is found in article, accounting more than half of the total output. Conference papers cover 34% of the total contribution, while book chapters and reviews contribute 3% and 5%, respectively. The limited representation of HRO in book chapters may be attributed to the relatively low emphasis placed by scholars on exploring alternative approaches to human resource management, such as HRO. The significant presence of HRO in articles can be attributed to the growing recognition and consideration of its effectiveness and efficiency as a unique approach to human resource management.

#### 4.1.5 Publications by citations

With a view to analysing the influential authors in the area of HRO, the present study considers the citations of the papers. Table 2 presents the top 10 most cited papers in the Scopus database as of October 2021. It is important to notethat the citation counts provided may differ slightly from those in ISI Web of Science or Google Scholar. The data presented in Table 2 highlights that the paper by Gilley et al. (2004) has received the highest number of citations in the HRO field, followed by Gospel and Sako with 70 citations. Additionally, Yu et al. (2016) and Cagliano et al. (2011) have received around 40 citations.

**Table 2:** Top ten cited papers in the HRO literature.

Authors	Title	Year	Citations
Gilley K.M., Greer C.R., Rasheed A.A.	Human resource outsourcing and organizational performance in manufacturing firms	2004	105
Gospel H., Sako M.	The unbundling of corporate functions: The evolution of shared services and outsourcing in human resource management	2010	70
Yu D., Zhang W., Huang G.	Dual hesitant fuzzy aggregation operators	2016	41
Cagliano A.C., Demarco A., Rafele C., Volpe S.	Using system dynamics in warehouse management: A fast-fashion case study	2011	40
Pereira V., Anderson V.	A longitudinal examination of HRM in a human resources outsourcing (HRO) organization operating from India	2012	30
Chiang F.F.T., Chow I.H.-S., Birtch T.A.	Examining human resource management outsourcing in Hong Kong	2010	27
Klaas B.S., Gainey T.W., McClendon J.A., Yang H.	Professional employer organisations and their impact on client satisfaction with human resource outcomes: A field study of human resource outsourcing in small and medium enterprises	2005	25
Irwin K.C., Landay K.M., Aaron J.R., McDowell W.C., Marino L.D., Geho P.R.	Entrepreneurial orientation (EO) and human resources outsourcing (HRO): A “HERO” combination for SME performance	2018	21
Tremblay M., Patry M., Lanoie P.	Human resources outsourcing in Canadian organisations: An empirical analysis of the role of organizational characteristics, transaction costs, and risks	2008	21
Schlosser F., Templer A., Ghanam D.	How human resource outsourcing affects organizational learning in the knowledge economy	2006	19

#### 4.1.6 Most common words appeared in the title

**Fig. 2:**
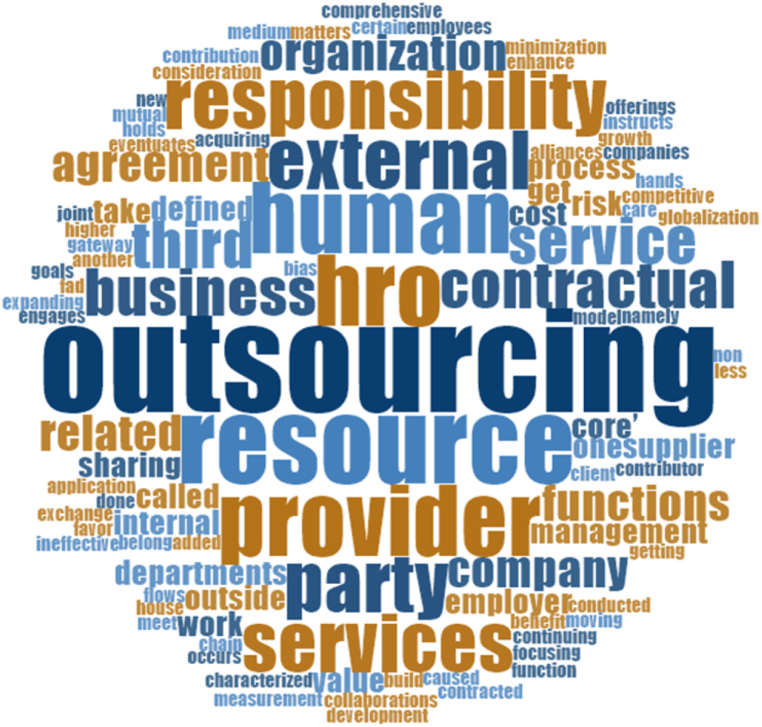
Word Cloud for the most common words in HRO publications.

To identify the most common words appearing in the titles of HRO publications, the word cloud feature of NVivo 12 (latest version) software was utilized. Table 3 presents the most frequent words found in HRO titles, including ‘outsourcing’, ‘resource’, ‘HRO’, and ‘human’. Fig. 2, generated from NVivo 12, visually presents these common words with more comprehensive and bold fonts, while relatively less frequent words are displayed in smaller fonts. Previous studies have argued the effectiveness of word clouds in exploring prevalent words in complex environments, commonly used in literature to identify prominent terms, keywords, and common themes in publications [[39], [40], [41]].

**Table 3:** Most common words used in the tittle.

Word	Count	Word	Count
Outsourcing	9	External	5
Resource	7	Party	5
HRO	6	Responsibility	5
Human	6	Services	5
Provider	6	Business	4

#### 4.1.7 Publications by subject area

As HRO is a branch of human resource management practice, it is practiced and accepted in several sectors and contributed to academic areas. As a result, the maximum contribution of 54% is made on business, management, and accounting, and it is more than half of the total. Further, the second-largest performance is seen in computer science, and it is 26%. Also, HRO practices are seen at 23%, 14%, and 12% in Engineering, Social Sciences, and Decision Sciences, consecutively. Additionally, Economics, Econometrics, and Finance cover 10% of the papers, while Mathematics accounts for 7%. Materials Science and Psychology both contribute 6% each, and the lowest percentage comes from Chemistry, representing 4% of the total.

#### 4.1.8 Publications by affiliation

HRO is predominantly practiced and has seen significant academic contributions from Asian countries. Among them, Tianjin University of Technology and the University of Malaysia Perils have contributed 5 papers each. Additionally, the University of Utara Malaysia and College of Business, University Utara Malaysia, have contributed 8 papers and 4 papers respectively. The lowest contribution, consisting of 3 papers, comes from the University of Sains Malaysia. The remaining papers are affiliated with a single institution.

## 5 Insights of HRO

The following subsections present the insights of 32 papers selected from the ISI web of science database.

### 5.1 Conceptual and theory development

This subsection intends to classify articles associated with the concept development and progression of theories in HRO. Initially, an integrated definition of HRO is is developed by examining definitions suggested by numerous studies in the literature. A comprehensive literature review has identified a list of 12 definitions from numerous sources, which is presented in Appendix-A. Using NVivo 12 software, the most similar words and ideas used in previous HRO definitions were explored through text searches. This search helped recognize the placement of similar words within sentence structures. The outcomes of these searches are presented in Table 3 and Fig. 2.

**Fig. 3:**
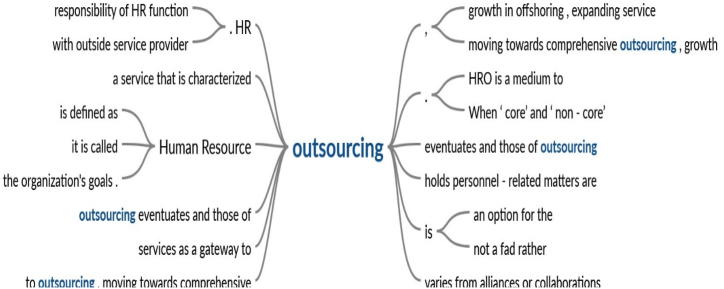
The placement of the word “outsourcing” in the definition

Based on the search results, the word ‘outsourcing’ appears most frequently, being used in 9 times out of the 12 definitions. To further explore the central theme of the concept, a text search using the word ‘outsourcing’ was conducted, and a ‘word tree’ was generated based on this specific word, as illustrated in Fig. 3.

Fig. 3 displays the ‘word tree’ associated with the past definitions, illustrating how the word ‘outsourcing’ is positioned within the sentence structure while defining HRO. For example, the phrases ‘responsibility of HR function,’ ‘with outside service provider,’ and ‘moving towards comprehensive outsourcing’ emerge as the most common contexts derived from prior definitions.

Moreover, shared responsibility, understanding the client's expectations, and the organization's capability, benefits, and risks are essential elements of the HRO process and crucial for successful outsourcing. However, top management's role in pursuing HRO success is often not strongly emphasized, and the responsibility for HRO implementation is commonly delegated to other employees [13]. Additionally, the presence of trust and close supervision is a core issue, as the absence of trust can disrupt information flow, create coordination problems, and lead to conflicts between organisations Again, provider quality and knowledge sharing are crutial factors that can be evaluated based on the recommendation from in-house personnel of the receiving company [8,13]. It is important to acknowledge thatthe HRO process involves multiple parties, including the firm's HR staff and managers of HR outsourcing service providers [9]. Taking the preceding discussion into consideration and focusing on the central theme, the following definition is proposed.HR Outsourcing involves the service receiver company delegating both core and non-core HR functions to an outsourcing company, aiming to achieve organizational and economic advantages by reallocating significant time and resources from employee management programs. The provided definition encompasses the involvement of HRO partners, such as vendor companies and inter-organizational employees, with an emphasis on trustworthiness and reliability.

In the evaluation of the HRO concept, previous studies have highlighted how it has emerged as a trusted partner for businesses. The service receiver company is not merely seekingthe right provider, but also a longstanding and reliable partner reliable partner. As a result,the evolution of HRO literature has embraced the contributions of numerous researchers, leading to a wide range of studies focused on the conceptual development of HRO. These studies are expanding rapidly, indicating the growing interest in this field. Furthermore, with regard tothe rapid growth of HR outsourcing decisions, researchers have shifted their focustoward evaluating thecontinuity of such decisions rather than the initial adoption of HRO [42]. The early researchers primarily examine the impact of HRO on manufacturing and SMEs firm performance [23]. Subsequently, an essay delves into examining the appropriateness of purchasing decisions of HR functions under specificcircumstances. For example, this includes assessing the suitability of HRO in the public sector [6], examining the impact of centralization and decentralization on the success of HRO initiatives [43], considering the availability of in-house resource resources [44], and evaluating the influence of firm'sstrategy and structure [45]. Apart from these, in addition to measuring both positive and negative effects, researchers also inquire about the factors that contribute to the outsourcing decisions [13,21,46]. In fine, research in this field continues to thrive and expand on a daily basis, which highlights the significance of conducting this study.

### 5.2 Drivers and drawbacks of HRO

Cost reduction and several other factors are often determine whether a company will engage an external vendor. Additionally, internal operational needs, resource limitations, urgency, and internal capabilities are taken into consideration.Traditional companies typically retain their HR department while attemping to save costs through economies of scale, lower technology-investment costs, efficient HR processes, and reduced headcount. In such circumstances, HRO rapidly copes with the new economic environment by providing cost savings [7] and gaining a competitive advantage [47].

Earlier studies by Pandey and Quartey [1,48] reveal that HRO impacts organisational learning both positively and negatively. However, beyond formal organisational boundaries, it provides a new learning network and offers specialized practice for a function, which is then obtained by the client company. Additionally, HRO becomes a medium to convert knowledge from tacit to explicit [1]. Furthermore, firms that engage in innovative products and aim to exploit the market are more likely to buy routine HR functions, while those that maintain a pace strategy are less likely to outsource non-core HR activities [4].

Therefore, HRO encompasses distinct quality and insights, taking a holistic approach that emphasises innovation and adaptability in new and pristine conditions [49]. Generally, firms outsource functions to allocate more time to their core business activities, rather than wasting time and energy on HR functions alone [50]. Along with the aforementioned factors, firms are influenced to buy HR functions because it provides access to new technology, minimizes risk, and eliminates internal biases within the organisation [21]. Following the employee retention concept, specialized training opportunities and succession planning foster social capital, which is firm-specific and encourages employees to stay with the organisation [51]. A study by Butler [5] found that outsourcing transactional HR functions can increase market share value, leading to positive cash flows, especially in service firms. However, in the long run, the return on investment may decline if firms failto optimize HRO participation.

Moreover, labor-intensive sectors that prioritize pragmatic and problem-solving tasks while maintaining service quality are increasingly practicing outsourcing HR functions to influence and regulate employee performance and behavioral resonance [49]. Decentralized firms find it effective to outsource their HR activities for standardizing their system and processes, rather than relying on a centralized firm due [43]. However, Table 4 presents the drivers related to HRO decision-making.

**Table 4:** List of drivers’ influence to take HRO decision.

Drivers	References
Cost advantage	[[5], [6], [7],^9^,^21^,^44^,^47^,[52], [53], [54], [55]]
Organizational Learning	[1,48,55]
Competitive advantage	[5,23,47,53]
Innovation	[4,44,56]
Opportunity to concentrate on core business functions and production boosting	[5,52]
Acquire new technology	[10,21]
Minimizing risk and preventing internal bias and data accuracy	[7,21]
Increase value of the market share with social support and relationship	[5,48]
Employee Retention	[51]
Employee performance and behavior resonant	[49]
Efficiency in warehouse management	[57]
Specialization by experts and technology	[7,43,44,54,55]
Customer satisfaction	[6,7]

More firms are engaging in HRO to strengthen their competitive positions, but it is not wise to depend solely on HRO programs. Instead, decisions should be made considering their strategic and economic positions [10]. Therefore, Table 5 presents some common drawbacks that HRO firms often face.

**Table 5:** List of drawbacks that happened by practicing HRO.

Aspect	Description	References
Lack of psychological contract among current employees	HRO leads to a reduction in the number of permanent employees and often necessitates the recruitment of seasonal employees. Consequently, this can create a gap in organizational and interpersonal relationships, leading to a lack of psychological contact and the dismantling of pre-existing networks and organizational memory. In the long run, the client company may experience inefficiencies, a lack of organizational values, challenges in developing tacit knowledge, and difficulty in obtaining unique competencies.	[1,58]
Risk of opportunist behavior	Despite the cost advantage of HRO, firms must exercise caution when using it due to the associated risk factors. Many firms fear outsourcing because they worry that the service provider might act as an opportunist.	[47]
Restrict Innovation	To gain a competitive advantage and enhance the firm's performance, both the prospectors and innovators avoid outsourcing core HR activities. Instead, they support in-house exercises.	[4]
Restrict to develop expertise	It is found that outsourcing core activities is not always fruitful; instead, it leads to a lack of expertise in the long run.	[9]
Risk of below-standard performance	The risk associated with performance and human resource development influences the decision of whether to make or buy the functions.	[46]
Labor Dispatch and turnover	Several developed and developing countries have adopted HRO and are thus involved in labor dispatch systems. This phenomenon creates a chaotic situation in labor markets, encouraging a lack of job security and psychological contact, and hindering both external and internal organizational culture.	[58]
Less customer Satisfaction	Organisations that outsource their executive recruitment and training tend to experience lower customer satisfaction, and vice versa.	[51]
Ignominy in Career Development	The impact of HRO varies depending on whether it is considered in the long-term or short-term time-bound, especially when it comes to the career development of an organization. A rising level of attrition, stress, and burnout necessitates thorough preparation by the administration for long-term career development.	[49]
Training Effectiveness	HRO for training programs often lacks effectiveness because managers find it challenging to identify the outsourceable components of training. Additionally, aligning the acquired knowledge with the organization's uniqueness becomes difficult, resulting in effectiveness challenges for the outsourced training program.	[23,45]
Misunderstanding regarding company culture	External vendors often struggle to grasp the uniqueness of firms, leading to a limited alignment with the firm's culture and characteristics.	[44]
Unusual Cost pressure	Despite being considered an effective driver sometimes, depreciation may also initiate a cost burden, as it may not be contingent on the market pay level.	[44]

### 5.3 Barriers to choose HRO practices

Outsourcing can be highly beneficial for an organisation, encompassing both core and non-core HR functions. However, the absence of an established guiding framework for the decision to outsource often leads to the discouragement of outsourcing core HR functions. This discouragement stems from various factors such as the organisation's strategy, structural choices, size, management style, as well as organizational fit and potential impact [4,21,49].

Following the trends, the large organisation intends to outsource HR related operational tasks and retains strategic responsibilities of HR activities [21]. Similarly, several other studies also highlight the potential loss of control in the implementation of HRO [13,44].

Moreover, HRO faces numerous challenges, such as the absence of proper management regulations and prior experience, difficulties in finding trustworthy suppliers, uncertainties regarding cost-effectiveness, and potential consequences in case of outsourcing failures [44].Perceived risk associated with HR outsourcing is also a common barrier to HRO adoption [46]. Moreover, HRO policies may face government regulation due to fears of labor dispatch [58]. Finally, it should be noted that the absence of a HR manager's commitment makesit unlikely for existing HRO practices to continue [42].

### 5.4 Functions outsourced

Despite the study of Chiang [44] suggesting the importance of maximizing internal resource utilization, organisations that have outsourced HR functions are likely to achieve a competitive advantage by considering HRO as a strategic practice [52]. However, not all outsourced functions have an equal impact, as they can affect the organisation's performance directly, indirectly, or simultaneously [52]. Additionally, it is important to note that not all service provider firms are the same; they have their own unique approaches.Some offer generalized services, while others specialize in specific HR functions [50]. In SMEs, only full-scale HR functions are typically outsourced, regardless of whether they are strategic or operational tasks [21].

Therefore, organisation often choose to outsource both core and non-core HRfunctions. Core HR functions that are commonly outsourced include recruitment and selection of managers, job development and salary grading, outplacement, employee appraisals, and HRM information systems [8]. On the other hand, non-core functions that are frequently outsourced include training and development, temporary staffing, organizational structure planning, personnel requirements, and the recruitment and selection of operational and support staff [8]. While non-core functions are typically the ones most likely to be outsourced [50]. Table 6 presents the circumstances of outsourced HR functions with a brief description.

**Table 6:** List of functions frequently outsourced in HRM.

Description	Reference
Recruitment, as a function of HR, directly impacts cost efficiency and the firm's performance, suggesting that managers should consider outsourcing recruitment functions.	[42,45,52,55]
Not only is payroll processing commonly outsourced, but it is also sometimes performed jointly with other companies, resulting in a positive impact.	[21,44,46,52,55]
Coaching and succession planning have a limited positive effect on a firm's performance.	[51]
Service organisations struggling with customer satisfaction are expected to outsource technology-linked HR activities, such as resume screening, annual benefits enrollment, etc.	[51,59]
Firms in pursuit of obtaining specific skills and capabilities intend to invest heavily in training and development. They may employ external trainers to offer a more extensive range of training and ensure higher quality that aligns with external market conditions.	[23,44,46,52,55,56]
It is discouraged to outsource HR planning, as well as compensation and reward systems.	[44]
A strategic HR department supports outsourcing its HR functions, specifically HRIS and labor relations.	[52,55]
Outsourcing benefits and payroll activities has been a comparatively old practice, but nowadays, HRO is performed as a full-service for organisations, helping them cope with overcapacity and outdated methods to control expenses.	[7]

### 5.5 A conceptual framework of HRO

**Fig. 4:**
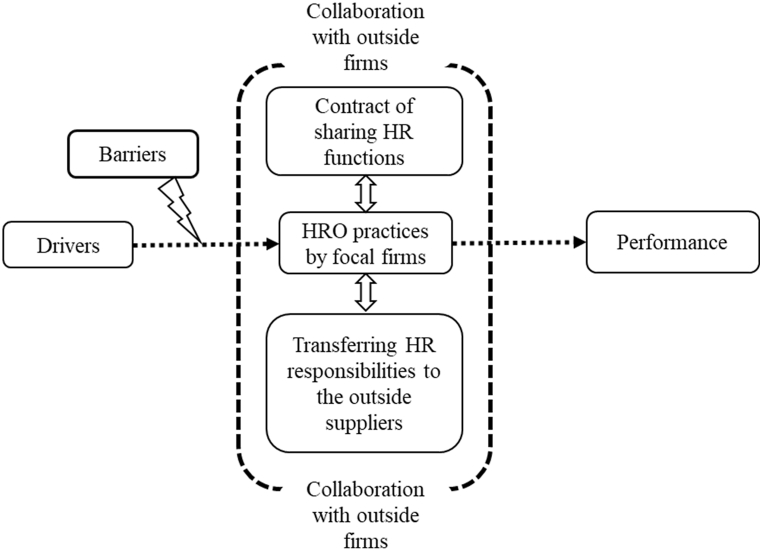
A HRO framework

The study contributes a comprehensive framework of HRO, which encompasses the drivers, barriers, and firm performance (see Fig. 4). Drivers are considered motivators influencing the focal firms to begin the practices of HRO. Previous researchers have argued that cost advantage is the most powerful driver of HRO [[5], [6], [7],^9^,^21^,^44^,^47^,[52], [53], [54], [55]]. Additionally other drivers are presented in Table 4, which positively motivate firms to adopt HRO. The robust presence of drivers results in faster adoption of HRO, as a lack of response to the driving factors in a timely manner might theaten the firm. On the contrary, some barriers might hinder the HRO practices. Ultimately, the more substantial and robust the barriers, the weaker the level of implementation of HRO.

The action firms regularly engage in HROas a part of their HR implementation strategy. Through HRO, these firms share their HR functions on a contractual basis, effectively transferring HR responsibilities to external suppliers. In other words, the focal firms delegate their HR tasks to outside suppliers. Therefore, HRO provides opportunities for fostering collaborative relationships with external firms.

Fig. 4 illustrates the drivers motivating the focal firms to adopt HRO practices. The arrow from drivers to HRO practices indicates a positive relationship between them. Additionally, successful adoption of HRO practices can lead to improved performance, represented by the right-side arrow from performance box. The barrier box with the arrow curving in the opposite direction represents hindrances caused by barriers to adopting HRO.

The outside dashed line indicates collaboration with external firms, helping focal firms adopt HRO. The inner boxes within the dashed line represent the HRO practices implemented by focal firms. However, the paper did not fully integrate all the drivers, barriers, and performance items into the comprehensive framework.Table 4, on the other hand, uncovers all the drivers that influence and underpin the adoption of HRO. Furthermore, Table 5, Table 6 present the drawbacks and functions outsourced by the focal firms, respectively.

## 6 Implications for academia and practitioners

### 6.1 Theoretical implications

This study has several theoretical implications. First, it offers an integrated and conclusive definition of HRO that can be used as a foundation for future research. This definition provides a clear understanding of what constitutes HRO and can help to ensure consistency in research across different studies. Second, the study identifies drivers, drawbacks, and barriers to the practice of HRO and the HR functions outsourced in an organisation. This can help researchers understand the factors that influence the adoption of HRO and its potential risks and benefits. Third, the paper highlights influential authors, top journals, and leading contributing countries, institutions, and disciplines in the field of HRO. This can help researchers identify the key players in the field and the most important sources of information. Fourth, the paper presents a simple, easy-to-understand framework of HRO that researchers can use to understand the critical elements of HRO practices in organisations. This framework provides a valuable tool for analyzing HRO practices and can help academia to understand effective HRO strategies. Finally, the study can guide future research in the field of HRO, providing insights into key trends, prospects, and challenges. Researchers and academicians can utilize this paper to identify gaps in current knowledge and explore new research directions, leading to advancements in the field of HRO.

### 6.2 Practical implications

The practical implications of this paper can benefit both organisations and practitioners involved in HRO. First, the study offers insights into the drivers and barriers to HRO adoption, which can help organisations make informed decisions when considering outsourcing HR functions. The study suggests that organisations can benefit from cost advantages and the ability to focus on core business functions, but they need to be aware of potential drawbacks, such as the risk of opportunism and the loss of psychological contact among current employees. Second, the study identifies recruitment, payroll processing, and technology-centric HR activities as the standard HR functions outsourced by organisations. This can help organisations decide which HR functions they can outsource to maximize the benefits of HRO. Additionally, the study presents a simple and comprehensive framework of HRO that can help practitioners understand the concept of HRO and apply it to their organisational context.

## 7 Conclusion

### 7.1 Research conclusion

Due to changes in the world's working ambiance and the way of working, there is an increased need to accomplish the tasks without physical presence. The pandemic situation and the mandate to limit social interactions have boosted the practice of HRO, encouraging both vendors and service receiver companies to keep their functions in top shape. Thus, HRO deserves more attention than a typical HR department. It is a new concept in which an organisation delegates core and non-core HR tasks as much as employee management requires. While the idea of HRO isn't new, the practice has gained popularity since the 21st century.

This paper identifies the reasons behind the rapid growth of HRO in connection with this idea. At the beginning of the study, we presented the concept of HRO by following a methodology that involves reviewing extant literature. In addition, the study systematically reviews and providess descriptive analysis based on metadata analysis and offers insights based on content analysis. We utilized a reliable database for data collection.

However, metadata analysis includes influential authors, funding and grant institutions, popular journals, publications by year, top contributing countries, institutions, document types, top-cited papers, and subject areas. According to the number of contributed papers, Abu Noor Hidayah was identified as the most influential author in this study. The International Journal of Human Resource Management and the Journal of Business Research are two of the most popular journals in this field. Furthermore, China emerged as the dominant country in terms of the number of published papers and their impact. Additionally, business, management, and accounting retain a significant share of the literature in HRO. To gain insight into the study, we considered the shortlisted papers from SCOPUS and Web of Science. Content analysis formed the basis of our insights, and we categorized the content into four sections: conceptual and theory development, drivers and drawbacks, barriers to choosing HRO practice, and outsourced functionsThe content analysis suggests that the author is still working on describing HRO in several dimensions, and as more reasons for the practice of HRO emerge, there are also many considerations to be taken into account. The contribution of this paper is to offer an integrated and conclusive definition of HRO. It presents key insights and trends in HRO, which significantly differ from earlier studies. The paper provides a simple, easy-to-understand, yet comprehensive framework of HRO. Ultimately, researchers and academicians can utilize this paper to explore future research directions while gaining a thorough understanding of the HRO concept.

### 7.2 Limitations and future research avenues

While the study provides valuable insights into the field of HRO, there are several limitations to consider. First, the study only considers papers published in the Scopus and ISI Web of Science databases, which may not comprehensively capture all relevant literature on the topic. Other databases and sources of information may have been excluded, potentially leading to a biased sample. Second, the study focuses on papers published from 2001 to 2021, which may not capture earlier research. As a result, important insights and trends from earlier years may have been missed. Third, the study only analyses papers written in English, which may exclude relevant research published in other languages. This may lead to a limited understanding of the global landscape of HRO research. Fourth, an objective approach to searching for data has been used instead of a subjective approach, which may lead to biased results in preparing a database. Fifth, to make it easy for general readers, the study confines its extent to metadata and content analysis. Sixth, the study relies on content analysis of papers, which may not capture the full scope of research on the topic. Other research methods, such as interviews or surveys, may provide additional insights not captured in the analysed papers. Therefore, while the study provides valuable insights into the field of HRO, these limitations should be considered when interpreting the results and implications for future research.

Some potential areas for further research might include: first, researchers could further explore the impact of HRO on various aspects of firm performance. While the study mentions that cost advantage, organisational learning, and the ability to focus on core business functions are drivers of HRO, it would be valuable to investigate how outsourcing affects factors such as employee morale, innovation, and customer satisfaction. Researchers could also examine the effects of HRO on different types of firms, such as startups, small and medium-sized enterprises, and multinational corporations.

Second, future research could investigate the role of technology in HRO. The study notes that technology-centric HR activities are commonly outsourced. Still, it does not delve deeply into how new technologies such as artificial intelligence and blockchain may be changing the landscape of HRO. Additionally, researchers could examine how companies use technology to manage outsourcing relationships and mitigate risks associated with HRO.

Third, researchers could further explore the impact of COVID-19 on HRO. It would be valuable to investigate how the pandemic has affected the practice of outsourcing HR functions. For example, researchers could examine whether companies are more or less likely to outsource HR functions in the wake of the pandemic and whether there are new opportunities or risks associated with HRO in a post-COVID world. Additionally, researchers could investigate how the pandemic has affected outsourcing relationships and how companies manage these relationships in a remote work environment.

Fourth, the research could delve into the ethical implications of outsourcing HR functions. This could include examining the impact of outsourcing on employee well-being, job security, and the potential exploitation of workers in outsourcing destinations. Researchers could explore ethical frameworks and guidelines for responsible outsourcing practices.

Fifth, this area of research could focus on understanding the governance mechanisms and risk management strategies organisations employ when outsourcing HR functions. It could examine how companies establish and maintain control over outsourced activities, mitigate data privacy and security risks, and ensure compliance with legal and regulatory requirements.

Sixth, researchers could conduct comparative studies to analyse different HRO models, such as full outsourcing, selective outsourcing, or shared services. This research could explore the advantages, disadvantages, and performance outcomes of each model, considering factors such as cost-effectiveness, flexibility, service quality, and strategic alignment.

Seventh, this area of research could focus on understanding the cultural and international dimensions of HR outsourcing. It could explore the challenges and opportunities of outsourcing HR functions across different cultures, countries, and legal systems. Researchers could investigate how cultural factors influence outsourcing decisions, the effectiveness of cross-cultural HR management, and strategies for fostering collaboration in international outsourcing relationships.

Given the growing importance of sustainability and corporate social responsibility, researchers could investigate how outsourcing HR functions align with sustainability goals. This research could explore the environmental and social impacts of HRO, including the carbon footprint associated with outsourcing activities, the treatment of workers in outsourcing destinations, and the adoption of sustainable practices within outsourcing contracts.

Finally, while many studies focus on the short-term outcomes of outsourcing HR functions, future research could examine the long-term effects. This could involve investigating the sustainability and durability of outsourcing arrangements, the impact on organisational culture over time, and the long-term implications for employee careers and skill development.

## 8 Production notes

### Author contribution statement

All authors listed have significantly contributed to the development and the writing of this article.

### 8.1 Data availability statement

Data will be made available on request.

### Additional information

No additional information is available for this paper.

## Declaration of competing interest

The authors declare that they have no known competing financial interests or personal relationships that could have appeared to influence the work reported in this paper.

## Figures and Tables

**Table:** 

Sources	Definition
[60]	HRO is a trend that belongs to shared services as a gateway to outsourcing, moving towards comprehensive outsourcing, growth in offshoring, expanding service offerings, focusing on process quality and measurement, and continuing globalization.
[61]	A phenomenon in which a company hands over a part of its in-house operations to a third party and builds a contractual relationship for HR services with an external provider is defined as Human Resource Outsourcing.
[60]	When core and non-core HR-related work are performed by external personnel rather than the company's regular employees, it is called human resource outsourcing.
[62]	HRO is a medium to obtain services from a third party or delegate partial or complete responsibility of HR function.
[61]	HR outsourcing is an option for companies that provides an opportunity to rid themselves of problems caused by ineffective HR departments and to exert pressure on them.
[5]	HRO occurs when a business instructs an external supplier to take responsibility and risk for HR functions and performs these tasks for the business.
[63]	HRO occurs when an organisation shares its human resource functions and transfers the responsibility of internal work to an outside supplier to obtain a specific service. It is characterized by one-way resource flows, from the provider to client, in exchange for predetermined favor, with typically no sharing of profits or mutual contribution. This distinguishes HRO from alliances, collaborations, or joint ventures.
[5]	A contractual agreement between an employer and an external third-party service provider whereby the employer transfers responsibility and management for certain HR, benefit, or training-related functions or services to the external provider.
[62]	HRO can be defined as a solution that enables human resource departments to obtain services with high added-value and reduced costs, thereby helping them maintain a competitive position. This process involvesentering into a contractual agreement with a third-party service provider.
[21]	HRO is a business model that offers potential solutions for cost considerations, specialization, acquiring new technology, minimizing risk, and mitigating internal bias through contractual agreements with external service provider.
[64]	Human resources outsourcing is not a fad but a tool to enhance the human resources value chain and foster the development of human resources as a business partner and strategic enabler in achieving business objectives.
[24]	HRO deals with personnel-related matters that are contracted to other organisations. It refers to the transfer of HR-centric responsibilities, including Human Resource Management functions (staffing duties), to external third parties.

## Appendix A

The following table shows the compilation of HRO definitions from different authors.
